# Supplementary material for: A porin-like protein used by bacterial predators defines a wider lipid-trapping superfamily
Source: Nat Commun. 2025 Jul 5;16:6213. doi: 10.1038/s41467-025-61633-0 (PMC12228718; doi:10.1038/s41467-025-61633-0)
Supplement: Supplementary file 1 — Supplementary Information [file 41467_2025_61633_MOESM1_ESM.pdf]

Supplementary Material

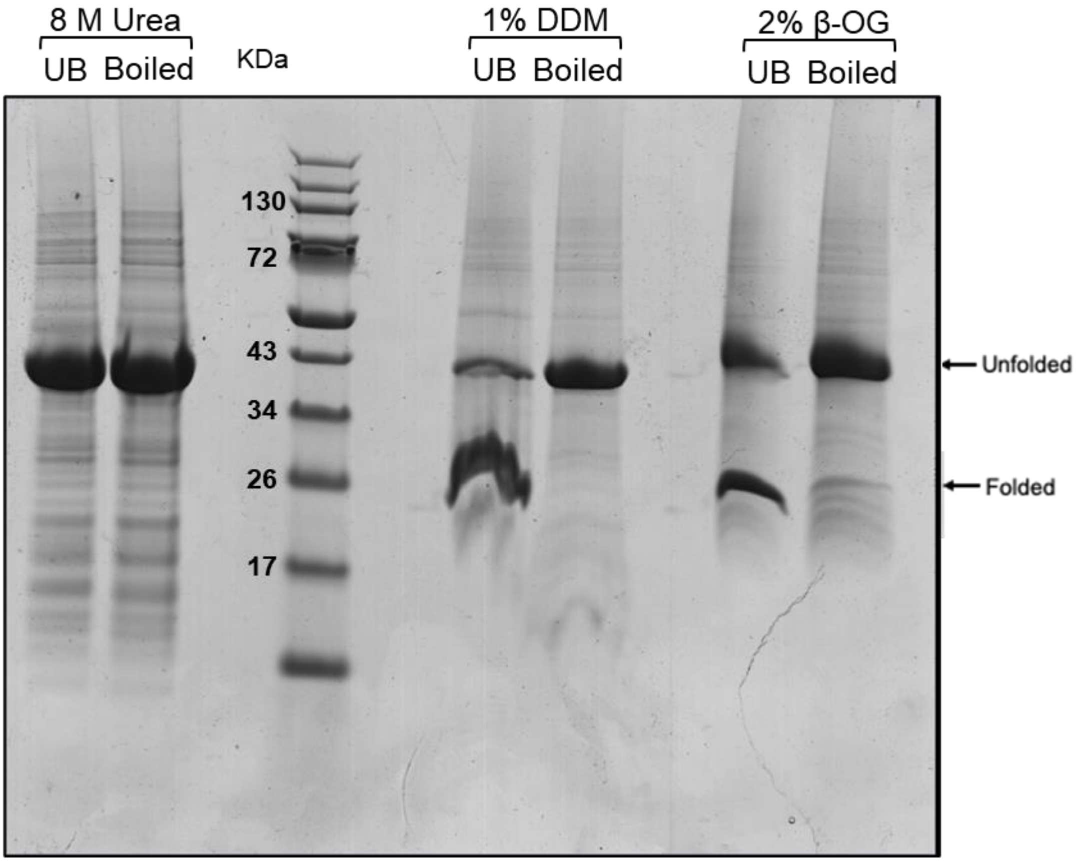

Supplementary Figure 1. Refolding heat-modifiability of PopA. Semi-native PAGE of PopA, showing samples boiled and unboiled (UB) in 8 M urea buffer, and those post dilution into refolding buffers containing either 1 % (w/v) DDM or 2 % (w/v) β-OG. Samples running further are classically designated as compact/folded.

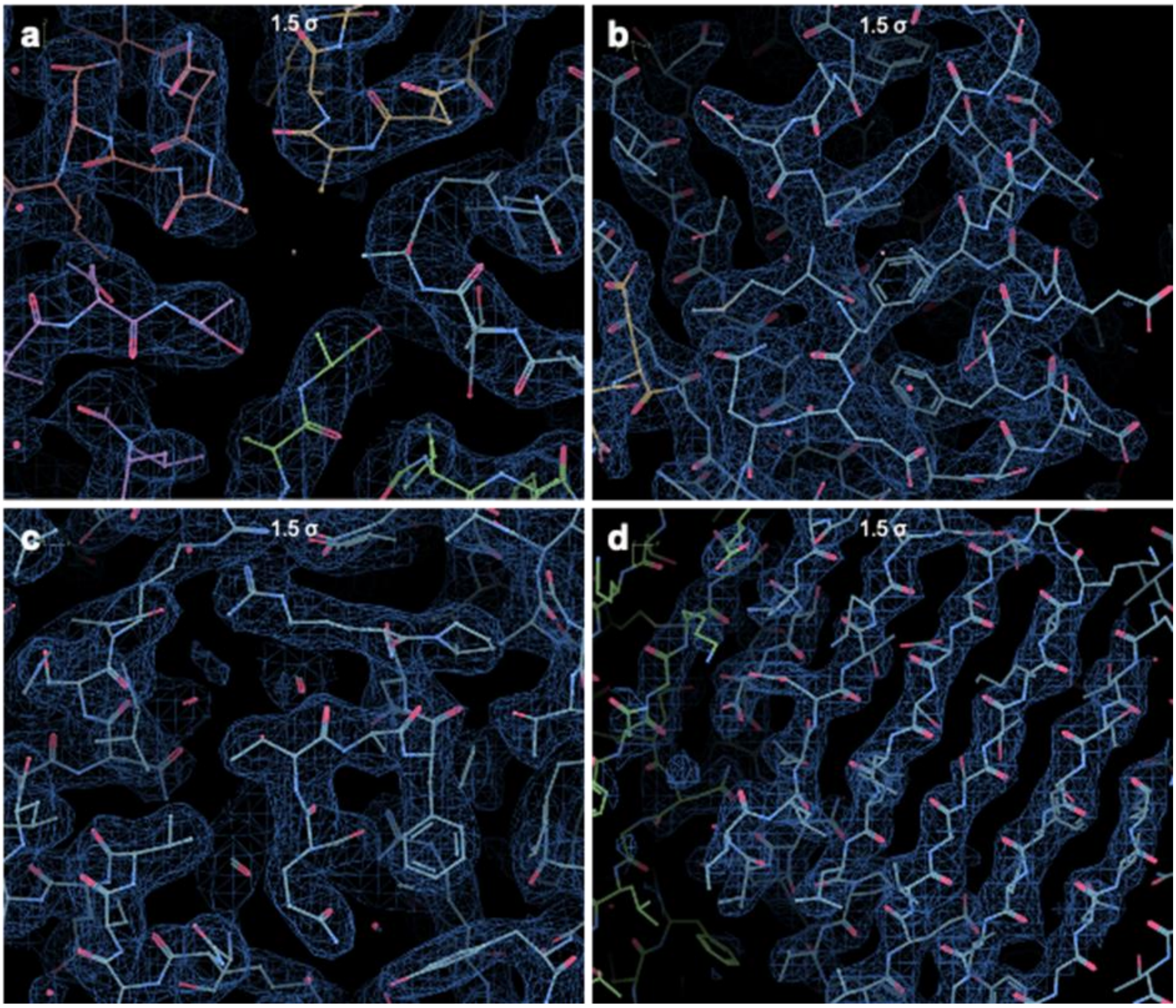

Supplementary Figure 2. 2$_{FO-FC}$ electron density map of PopA. Subpanels show representative electron density from several regions of the protein. a) The five-fold axis of the pentamer contributed by loop 8 from each monomer. b) The α-helix in loop 2. c) A region of the N-terminal plug. d) The β-strands on the exterior wall of the pentamer.

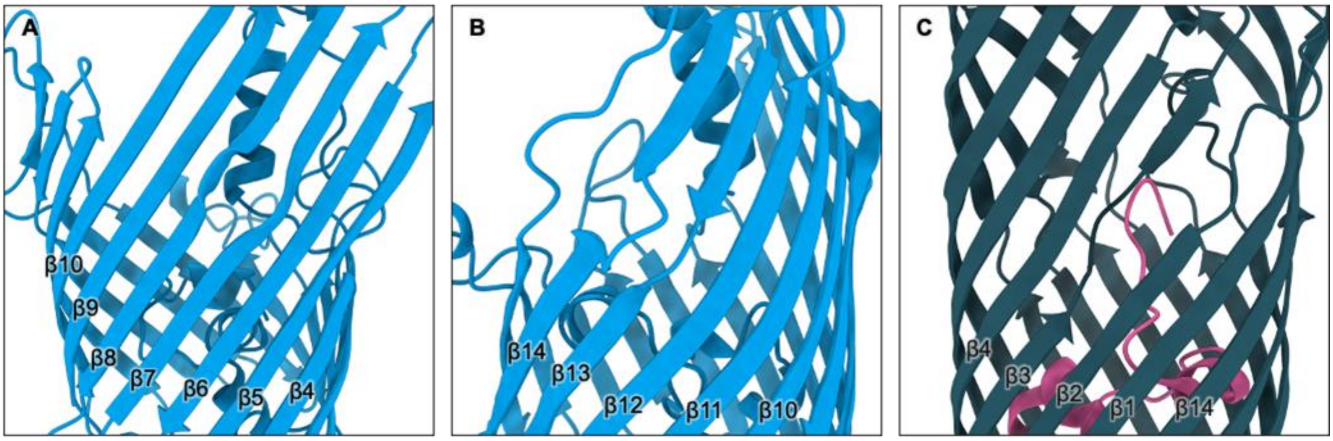

Supplementary Figure 3. Comparison of PopA and FadL sidewall openings. a and b illustrate the hydrogen-bond disruption in β6/7 and β13/14 of PopA; c illustrates the similar disruption in β2/3 of FadL.

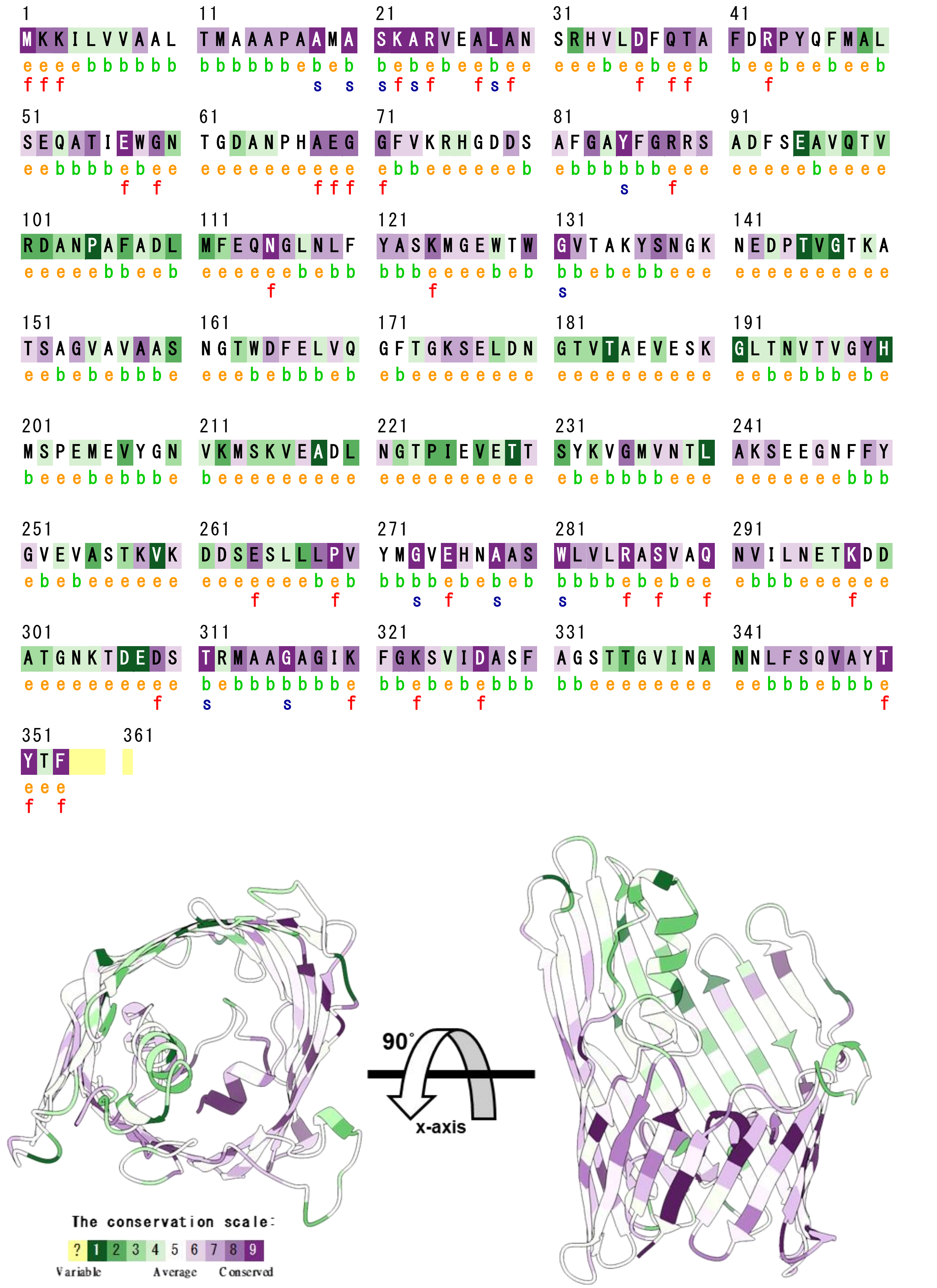

Supplementary Figure 4. Conserved residues of PopA, via ConSurf[23]. a) Sequence of PopA coloured to show conserved residues, f- predicted function residue, s- predicted structural residue. b) Cartoon of crystallographic structure with ribbon colouration matching conservation scale.

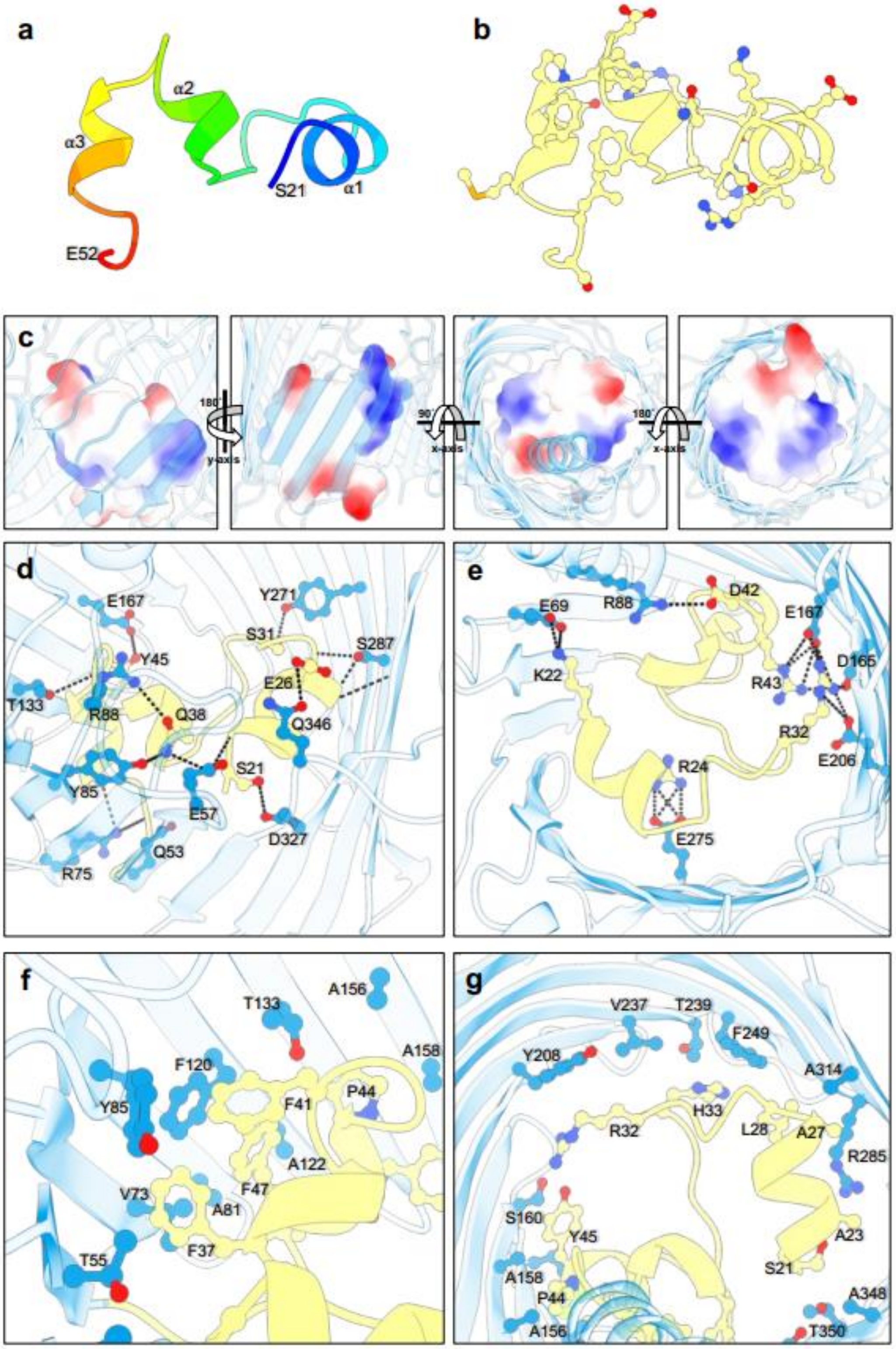

Supplementary Figure 5. The N-terminal plug and Interactions with the Barrel. a) Cartoon representation of the N-terminal plug of Bd0427, coloured in rainbow (blue – red). b) Cartoon diagram showing the residues involved in the plug barrel interface. c) Schematic of the electrostatic surface of the plug from four different orientations. d-g) Cartoon diagrams of residues involved in forming hydrogen bonds (d) salt bridges (e) and hydrophobic and van der Waals contacts (f, g) between the plug and the barrel wall.

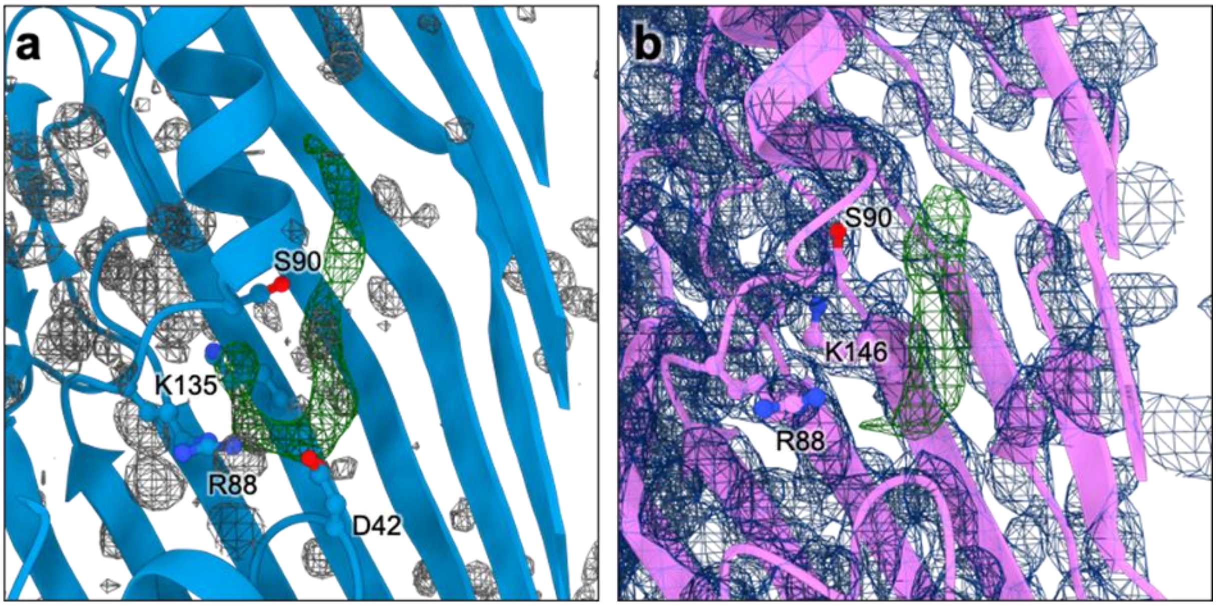

Supplementary Figure 6. Electron density behind loop 2. a) PopA crystal structure with mFo-DFc map (3.0 σ), with volume of positive difference density coloured in green. b) PopA cryo-EM structure in EM density map (map level 0.15) with unmodelled moeity coloured in green.

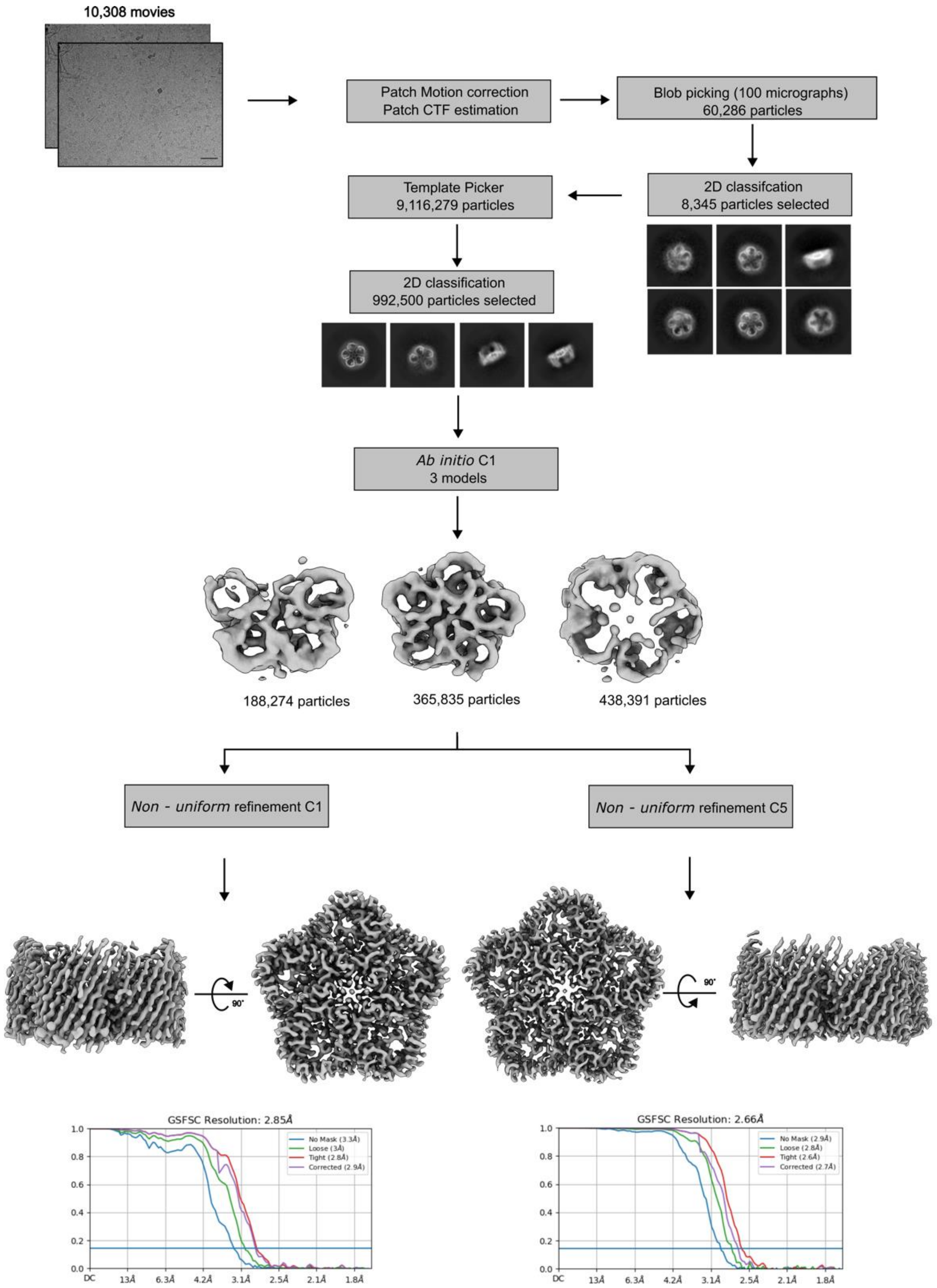

Supplementary Figure 7. Cryo-EM data processing workflow. Cryo-EM data processing within cryoSPARC v4.2.1[26]. Total of 10,308 movies were collected. Raw movies were processed using patch motion correction (multi), with dose weighting. The Contrast Transfer Function (CTF) was estimated by patch CTF estimation (multi). Particles were blob picked, 2D classes were inspected and used as a reference for complete particle picking. A total number of 992,500 particles were subjected to ab initio model generation. Three models were generated in C1 symmetry, one model (365,835 particles) was selected for further processing. The consensus map was refined with non-uniform refinement to a 2.85 Å resolution in C1 symmetry and 2.66 Å in C5 symmetry. The average resolution derived from Fourier Shell Correlation (FSC). FCS plot show correlation between two independent refined maps (half-maps) with blue (no mask), loose mask (red), tight mask (cyan) and corrected (purple). Cut-off 0.143 (blue line) was used for resolution estimation. Final maps are displayed at contour level 0.29.

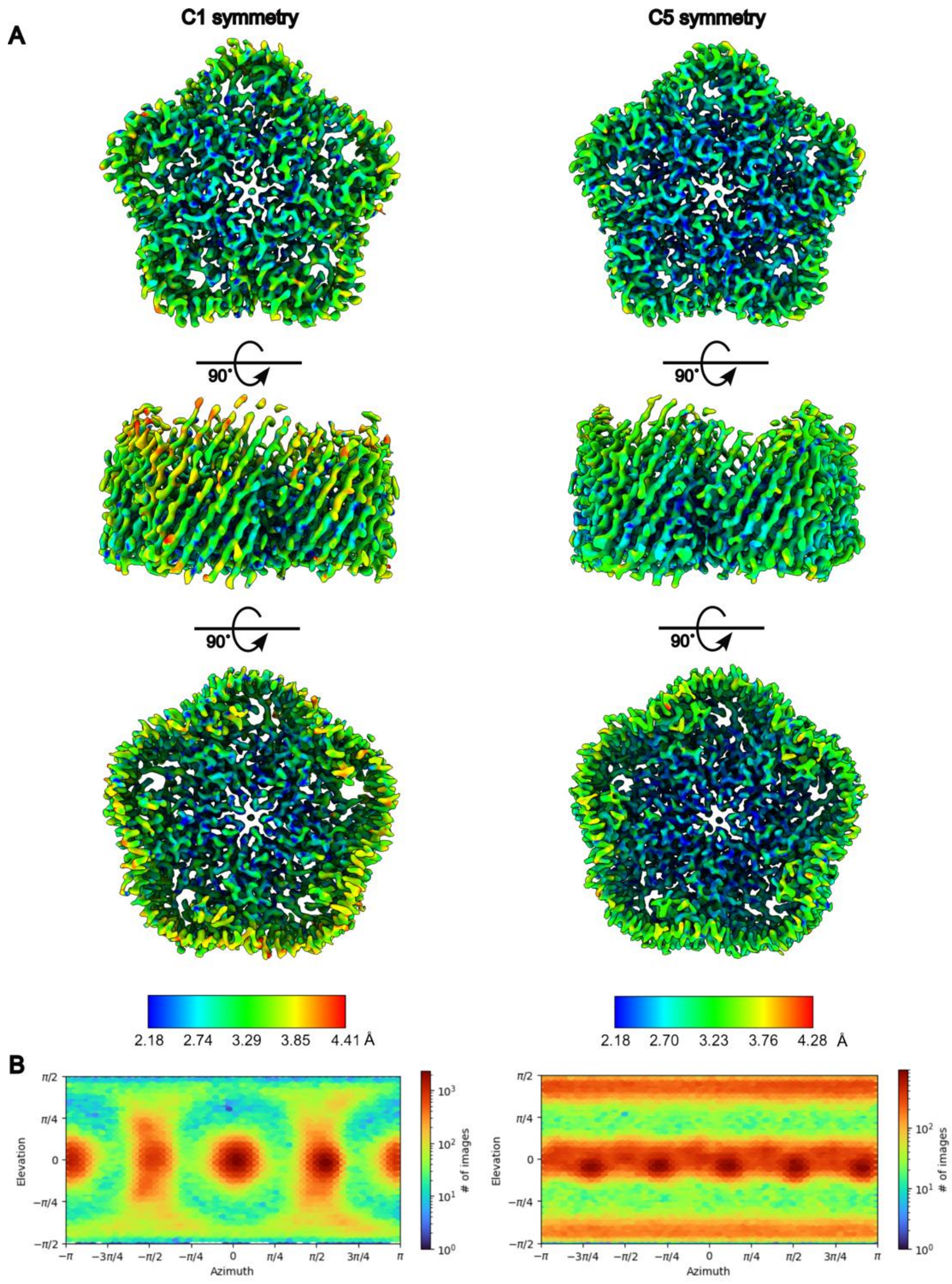

Supplementary Figure 8. Local resolution estimation. a) $PopA_{his}$ cryo-EM density maps in C1 and C5 symmetry, coloured according to local resolution. Local resolution was estimated using cryoSPARC v4.2.1[26] (FSC cut-off 0.5) and coloured as indicated by the scale below the map. Maps are displayed at a contour level 0.29. b) Final Euler angle distribution of the corresponding $PopA_{his}$ density maps.

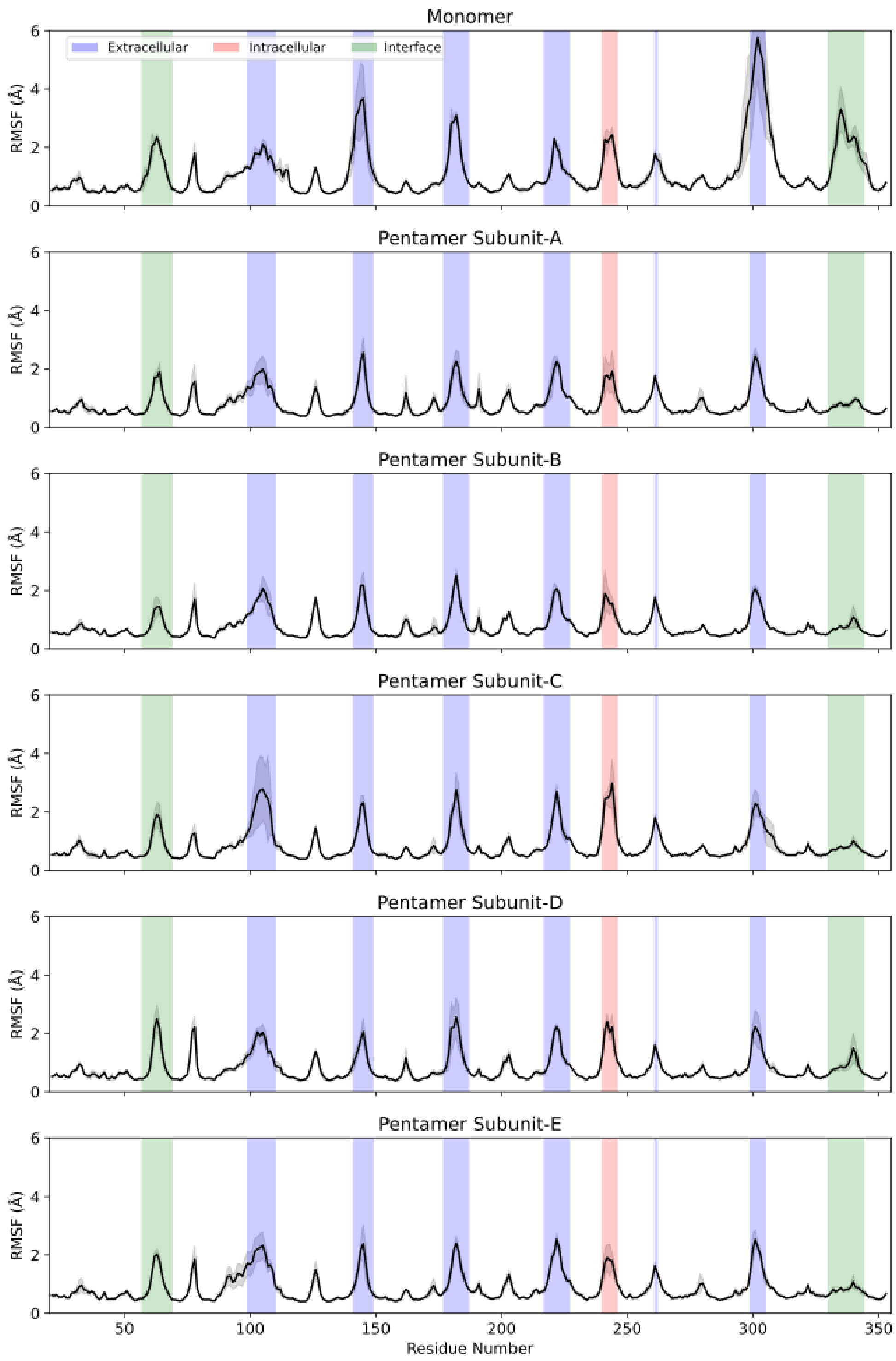

Supplementary Figure 9. RMSF of PopA loops in inner membrane. Root Mean Square Fluctuation (RMSF) values for loops in the PopA monomer and pentamer subunits embedded in the inner membrane, averaged over three simulation repeats. Green indicates loops at the protein interface, blue denotes extracellular loops, and red represents intracellular loops. The pentamer state has relatively dampened fluctuations compared to the monomer.

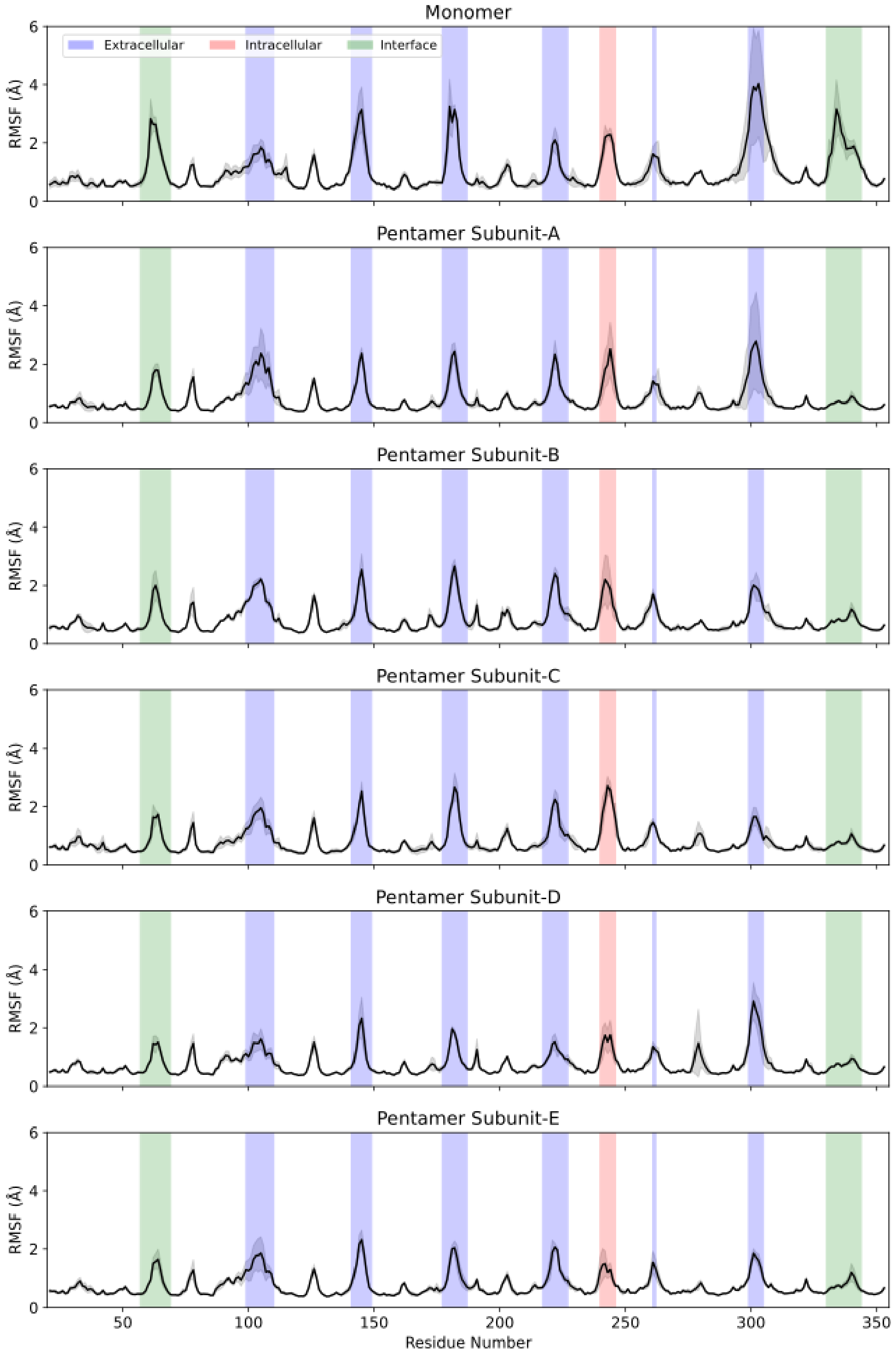

Supplementary Figure 10. RMSF of PopA loops in outer membrane. Root Mean Square Fluctuation (RMSF) values for loops in the PopA monomer and pentamer subunits embedded in the outer membrane, averaged over three simulation repeats. Green indicates loops at the protein interface, blue denotes extracellular loops, and red represents intracellular loops. The pentamer state has relatively dampened fluctuations compared to the monomer.

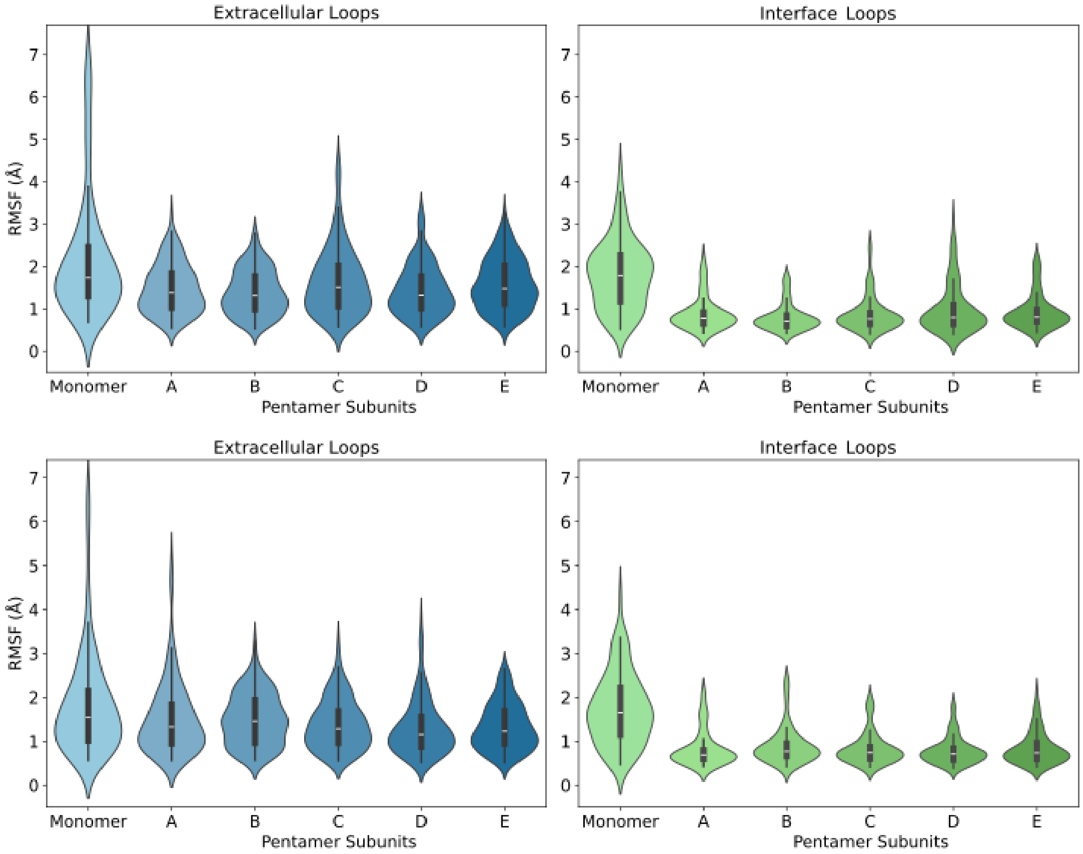

Supplementary Figure 11. Comparison of loop RMSF values in monomeric and pentameric models. RMSF values for PopA monomer and pentamer loops in the outer (top) and inner (bottom) membranes, showing the fluctuations of the extracellular (blue) and interface loops (green) across three repeat simulations, demonstrating decreased mobility in the pentameric state.

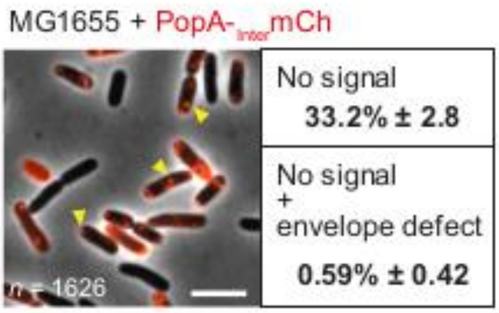

Supplementary Figure 12. Representative fluorescence microscopy of *E. coli* MG1655 producing the PopA-IntermCherry fusion protein. Examples of PopA-IntermCherry localisation are shown (yellow arrowheads). The percentage of cells that do not produce PopA-IntermCherry (No signal), as well as the percentage of cells that exhibit envelope defect without any signal (No signal + envelope defect) are indicated on the right. The number of analysed cells (n) is indicated below. Scale bar, 5 μm.

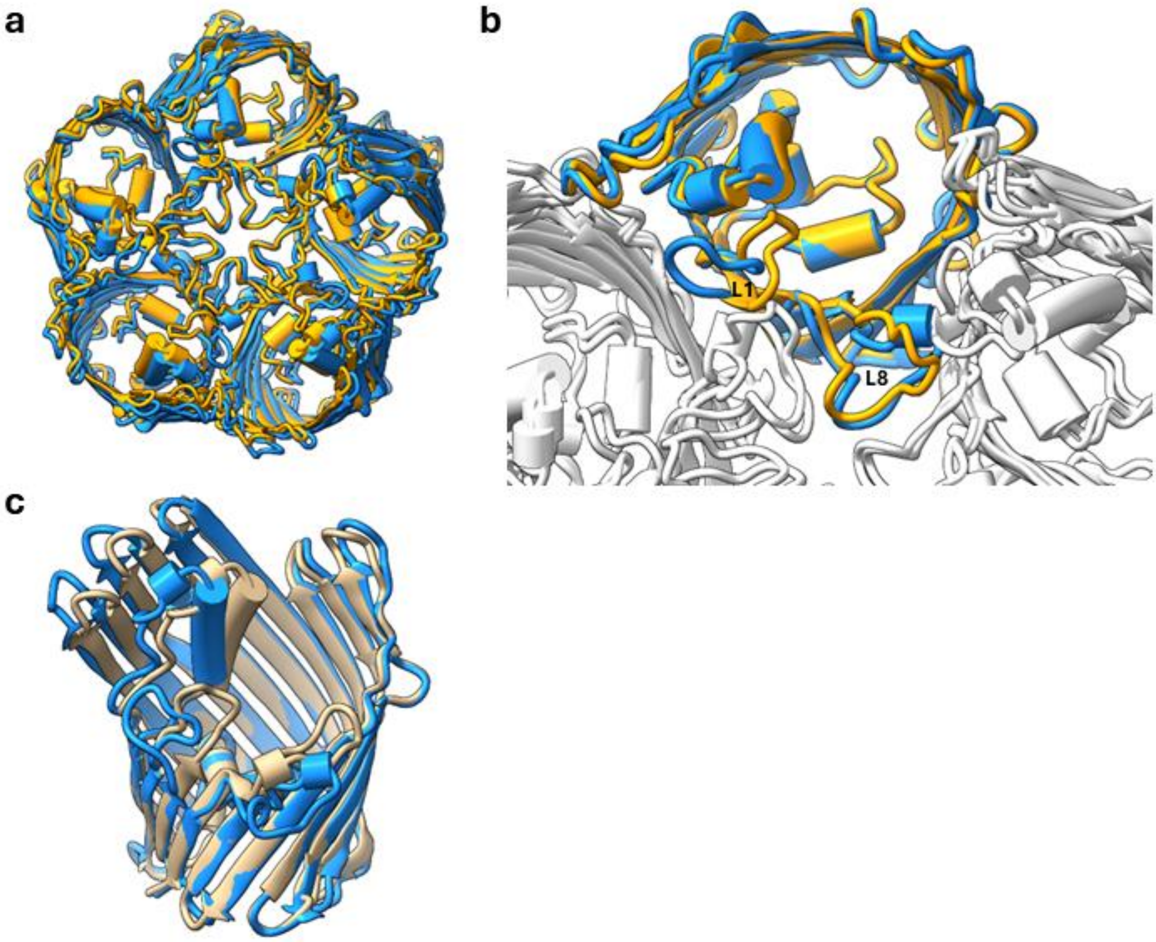

Supplementary Figure 13. Alphafold model of PopA. a) PopA experimental model (x-ray, blue) compared to Alphafold3[24] pentamer model (orange), aligned on chain A (uppermost monomer). b) zoomed-in view to demonstrate model differences at centre loops L1 and L8; L1 is in completely different pose and L8 lacks helical element. c) Comparison of x-ray model (blue) to Alphafold monomer model (beige).

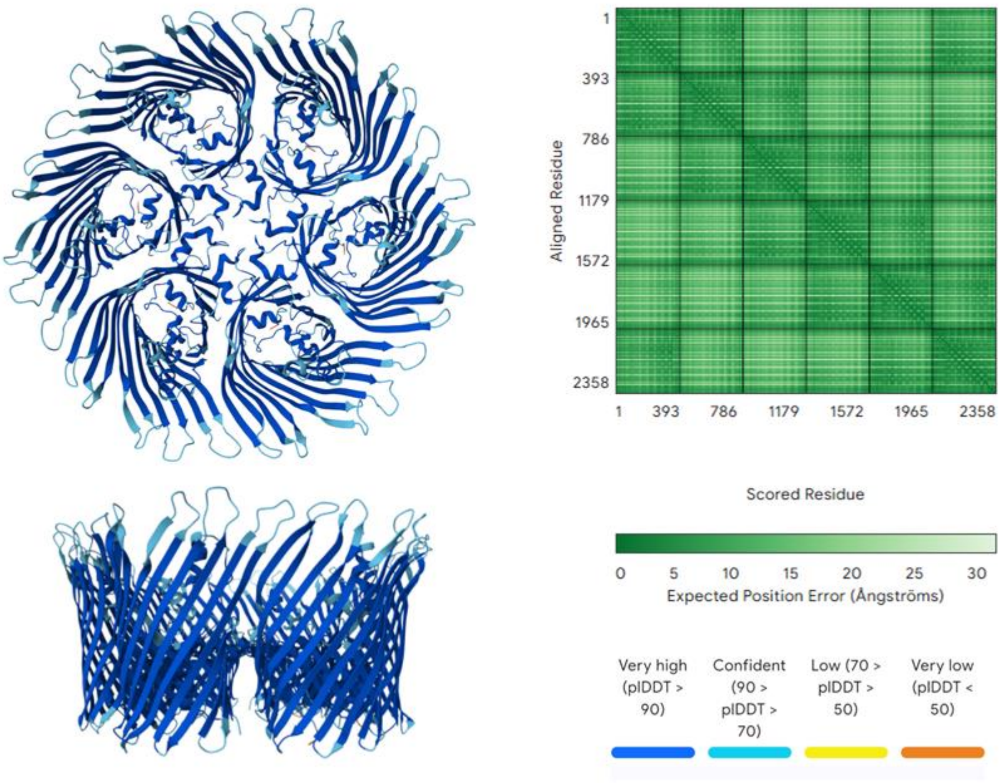

Supplementary Figure 14. Alphafold model of IPM56_03505 hexamer. Lefthand side two orthogonal views, coloured by pLDDT. Righthand side PAE (predicted aligned error) score matrix. Full stats and model available in ModelArchive (www.modelarchive.org/doi/10.5452/ma-n3ds4).

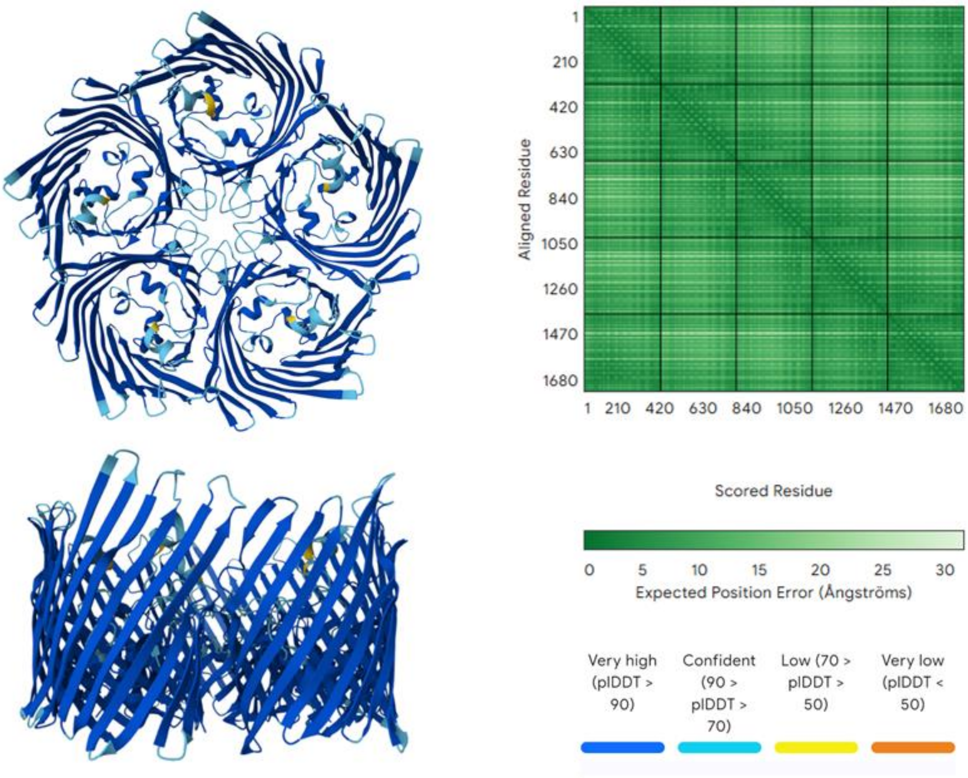

Supplementary Figure 15. Alphafold model of C0V70_02135 pentamer. Lefthand side two orthogonal views, coloured by pLDDT. Righthand side PAE (predicted aligned error) score matrix. Full stats and model available in ModelArchive (www.modelarchive.org/doi/10.5452/ma-e64a6).

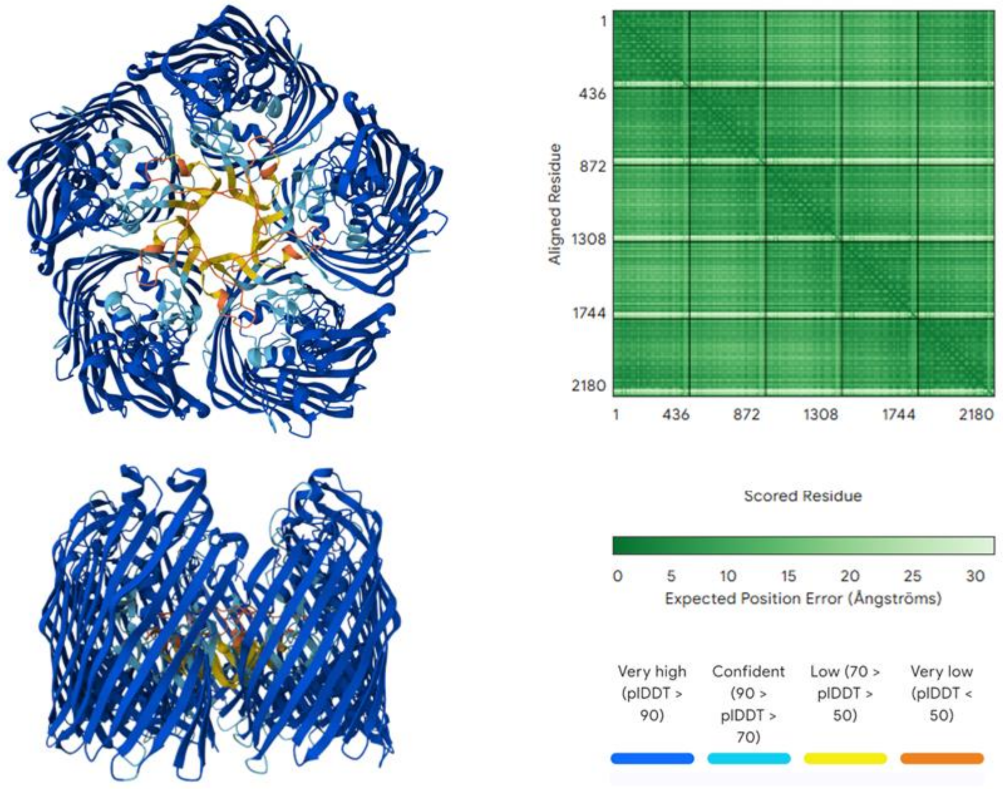

Supplementary Figure 16. Alphafold model of C0V70_02140 pentamer. Lefthand side two orthogonal views, coloured by pLDDT. Righthand side PAE (predicted aligned error) score matrix. Full stats and model available in ModelArchive (www.modelarchive.org/doi/10.5452/ma-uceig).

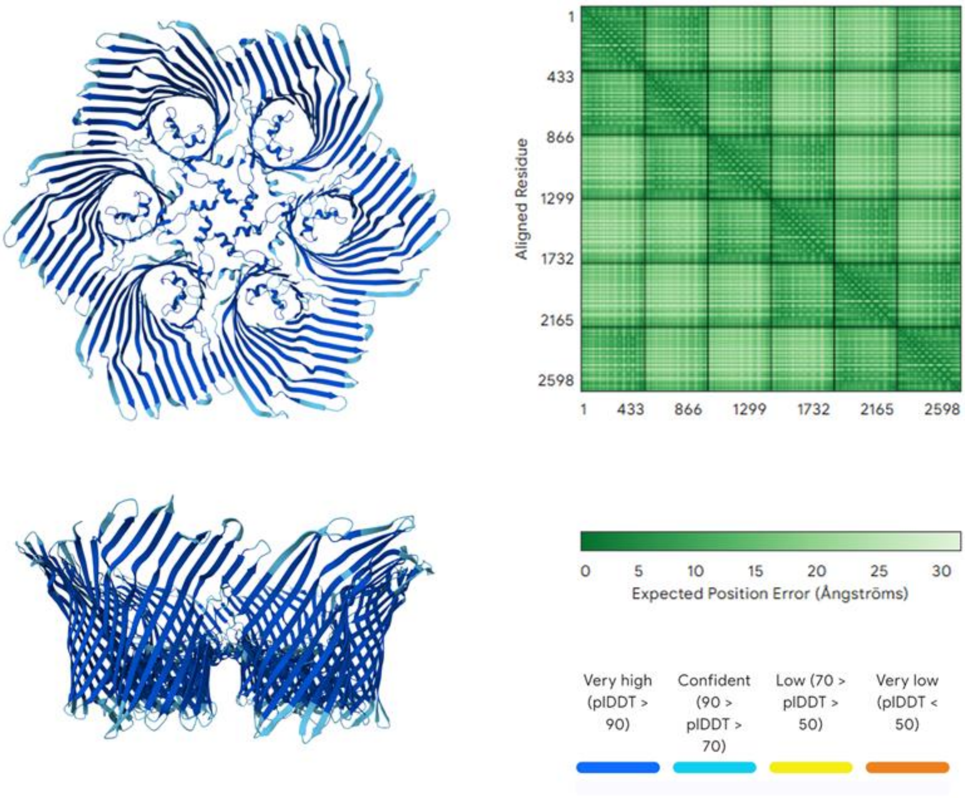

Supplementary Figure 17. Alphafold model of Tde_2508 hexamer. Lefthand side two orthogonal views, coloured by pLDDT. Righthand side PAE (predicted aligned error) score matrix. Full stats and model available in ModelArchive (www.modelarchive.org/doi/10.5452/ma-rkx5z).

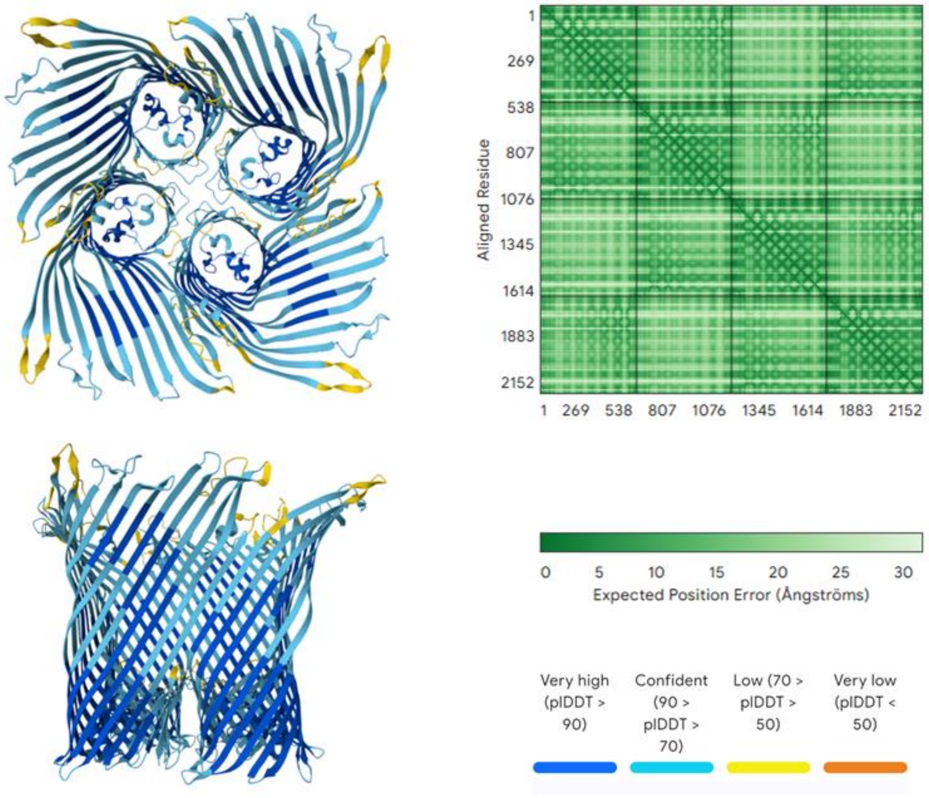

Supplementary Figure 18. Alphafold model of Hore_23180 tetramer. Lefthand side two orthogonal views, coloured by pLDDT. Righthand side PAE (predicted aligned error) score matrix. Full stats and model available in ModelArchive (www.modelarchive.org/doi/10.5452/ma-lgpuq).

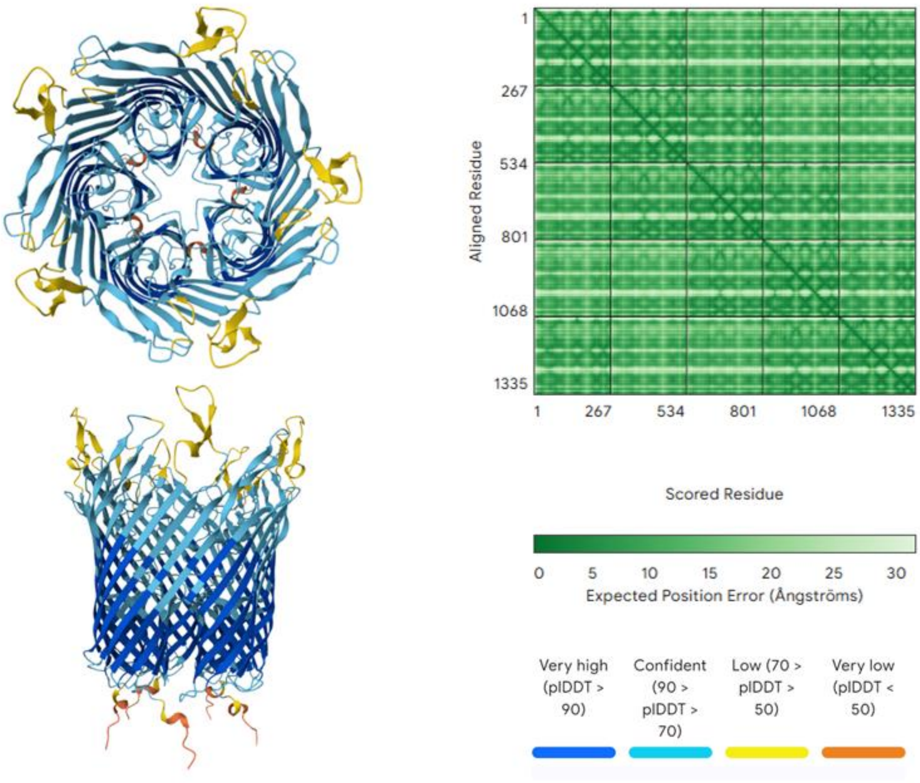

Supplementary Figure 19. Alphafold model of Bt_1926 pentamer. Lefthand side two orthogonal views, coloured by pLDDT. Righthand side PAE (predicted aligned error) score matrix. Full stats and model available in ModelArchive (www.modelarchive.org/doi/10.5452/ma-c773r).

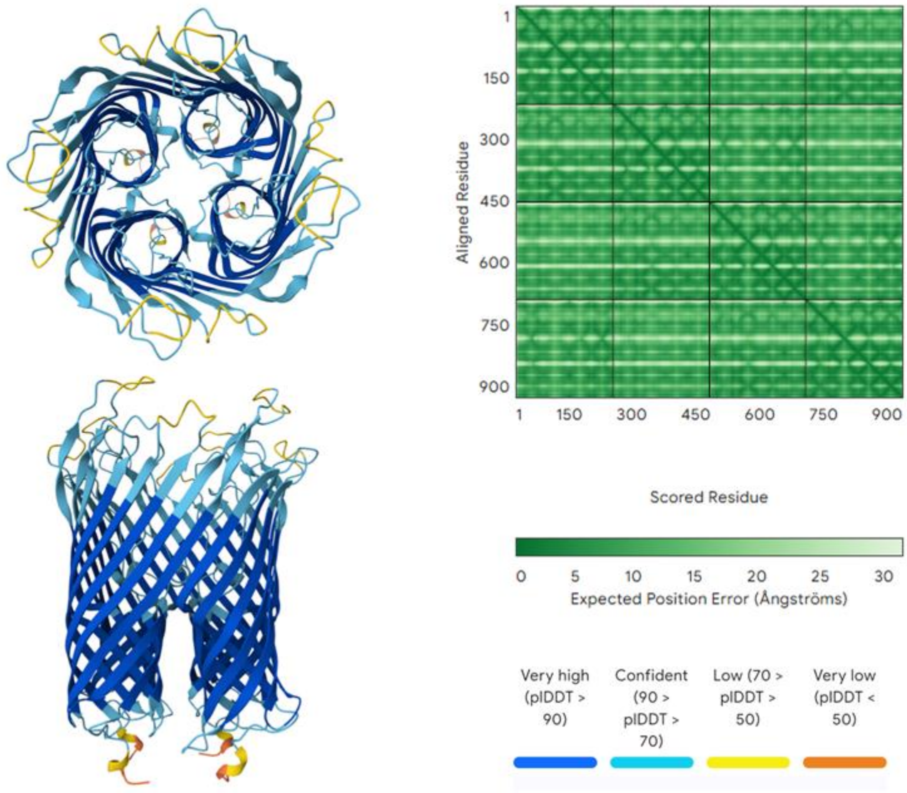

Supplementary Figure 20. Alphafold model of HMPREF9455_00941 tetramer. Lefthand side two orthogonal views, coloured by pLDDT. Righthand side PAE (predicted aligned error) score matrix. Full stats and model available in ModelArchive (www.modelarchive.org/doi/10.5452/ma-zp266).

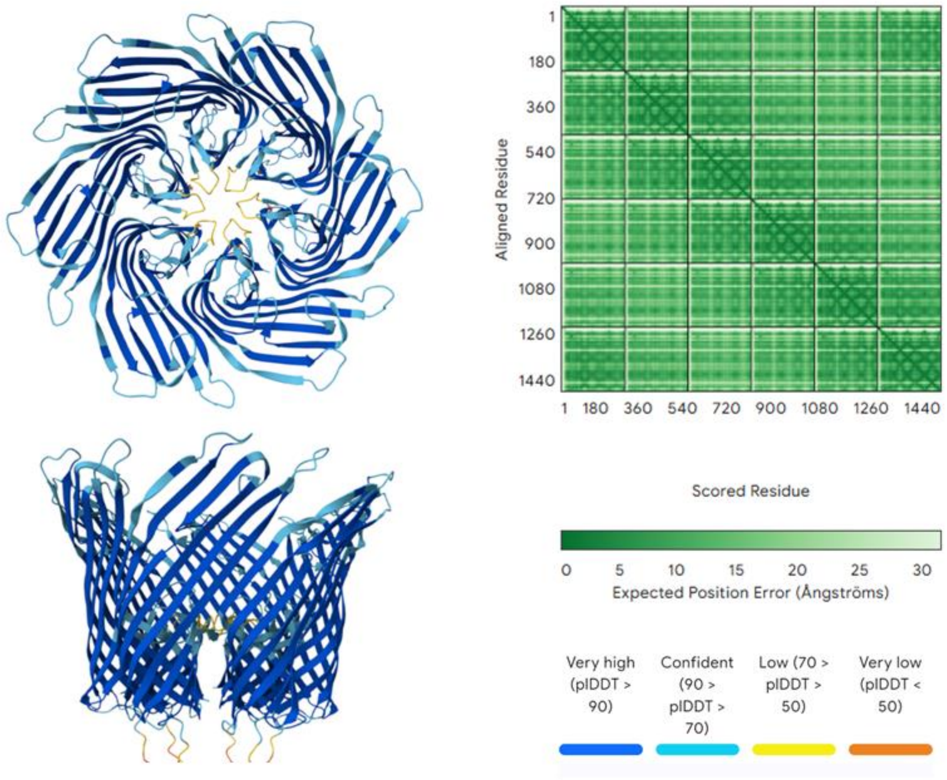

Supplementary Figure 21. Alphafold model of CHU_0007 hexamer. Lefthand side two orthogonal views, coloured by pLDDT. Righthand side PAE (predicted aligned error) score matrix. Full stats and model available in ModelArchive (www.modelarchive.org/doi/10.5452/ma-czwi5).

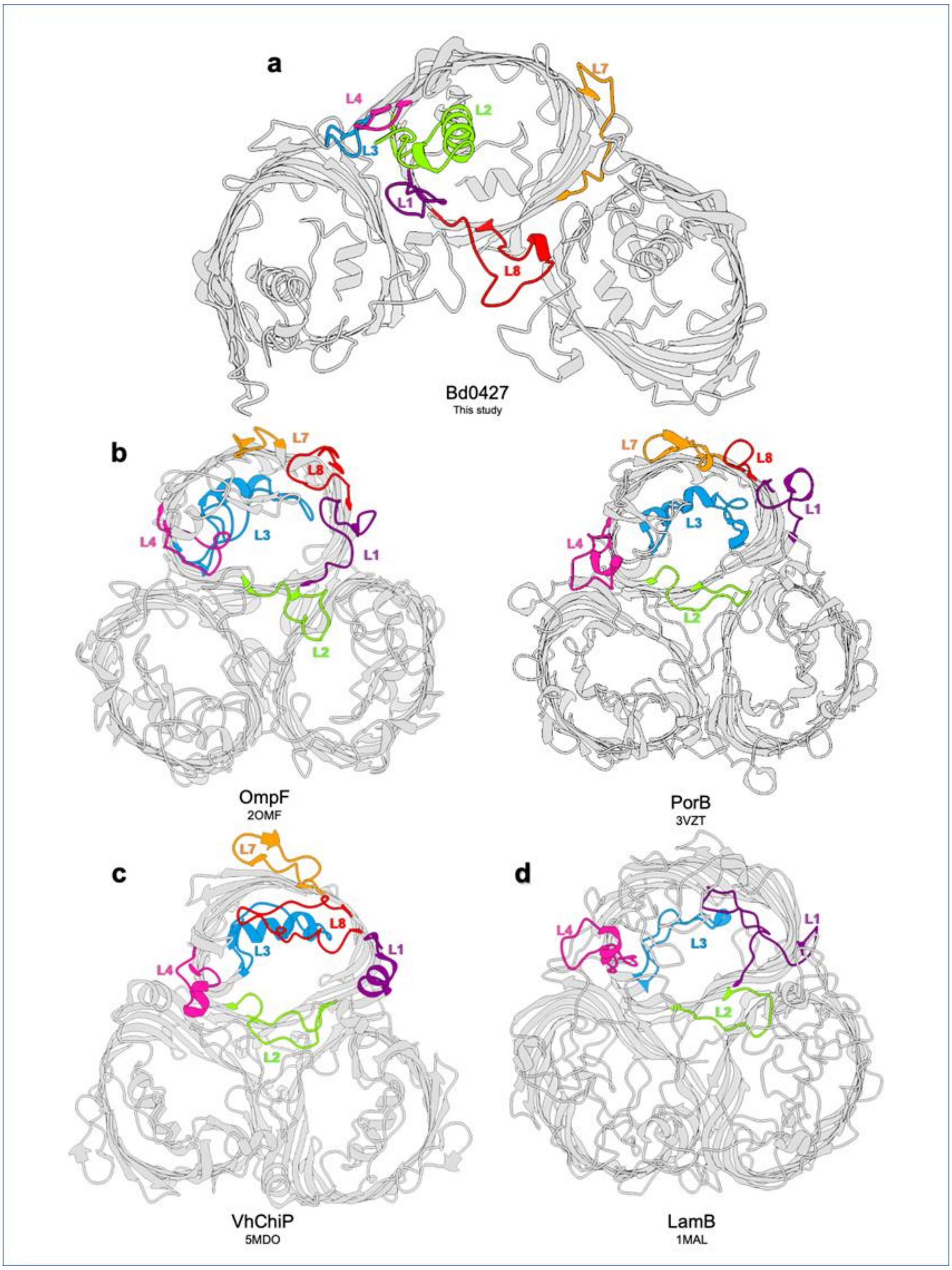

Supplementary Figure 22. Comparison of PopA/Bd0427 loops to Homotrimeric Porins and Specific Channels. a) PopA/Bd0427 structure with loops involved in homopentamer interface and those that form homotrimers in other published structures coloured as individual elements. b&c) Structure of three 16-stranded β-barrel proteins (two porins (b) and a specific channel (c) and the loops involved in forming a homotrimer/homopentamer coloured. d) Structure of 18-stranded specific channel with the loops involved in forming the homotrimer coloured, along with loop 3; loops 7 and 8 are not coloured as these cannot be directly compared due to change in number of strands forming the barrel.

Supplementary Table 1. X-ray crystallography data collection details.

	PopA (Bd0427)
**Data collection**	
Space group	C 2 2 21
Cell dimensions	
a, *b*, *c* (Å)	175.43, 191.35, 184.31
α, β, γ (°)	90, 90, 90
Wavelength (Å)	0.9794
Resolution (Å)	63.54-2.8(2.9-2.8) *
R_{sym} or R_{merge}	0.16(1.01)
I / σI	12.26(1.48)
Completeness (%)	99.89(99.96)
Redundancy	13.9(13.9)
**Refinement**	
Resolution (Å)	63.54-2.8(2.9-2.8)
No. reflections	76240(7545)
R_{work} / R_{free}	0.1994(0.3064)/ 0.2395(0.3827)
No. atoms	13197
Protein	12652
Ligand/ion	405
Water	295
B-factors	46.39
Protein	46.39
Ligand/ion	60.93
Water	40.22
R.m.s. deviations	
Bond lengths (Å)	0.008
Bond angles (°)	1.29

*Data collected from 1 crystal. *Values in parentheses are for highest-resolution shell.

Supplementary Table 2. CryoEM data collection details.

	PopA$_{His}$(Bd0427)<br>(EMDB-51308)<br>(PDB 9GF0)
**Data collection and processing**	
Magnification	105,000
Voltage (kV)	300
Electron exposure (e–/Å^2)	55.4
Defocus range (μm)	-2.4 to -1.2
Pixel size (Å)	0.835
Symmetry imposed	C1
Initial particle images (no.)	992,500
Final particle images (no.)	365,835
Map resolution (Å)	2.85
FSC threshold	0.143
Map resolution range (Å)	2.18 – 4.41
**Refinement**	
Initial model used (PDB code)	9GF0
Model resolution (Å)	2.85
FSC threshold	0.143
Model resolution range (Å)	2.18 – 4.41
Map sharpening *B* factor (Å^2)	106.1
Model composition	
Non-hydrogen atoms	12619
Protein residues	1657
Ligands	33
B factors (Å^2)	
Protein	42.74
Ligand	…
R.m.s. deviations	
Bond lengths (Å)	0.42
Bond angles (°)	0.58
Validation	
MolProbity score	2.3
Clashscore	9.3
Poor rotamers (%)	3
Ramachandran plot	
Favored (%)	94.14
Allowed (%)	5.68
Disallowed (%)	0.18
Model vs. data	
CC (mask)	0.87
CC (box)	0.71
CC (peaks)	0.69
CC (volume)	0.83

Supplementary Table 3. PopA superfamily members. Features of family members are given, alongside pTM/ipTM Alphafold3[19] scores for oligomer state models. Length of oligomer loop is presented next to likely oligomer state.

Gene	Grouping, species	Size	State	Tetramer	Pentamer	Hexamer
Bd0427	Bdellovibrionota; *Bdellovibrio bacteriovorus*	353	5-fold; 16aa	0.57/0.64	**0.85/0.86**	0.76/0.79
C0V70_02135	Bdellovibrionota; *Bacteriovorax* stolpii	356	5-fold; 21aa	0.56/0.64	**0.89/0.9**	0.82/0.84
C0V70_02140	Bdellovibrionota; *Bacteriovorax stolpii*	456	5-fold; 31aa	0.64/0.7	**0.86/0.88**	0.75/0.78
KC933_17480	Myxococcota; Unclassified	495	6-fold; 26aa	0.71/0.76	0.61/0.67	**0.82/0.84**
DN745_13755	Deltaproteobacteria; *Bradymonas sediminis*	431	6-fold; 20aa	0.69/0.74	0.7/0.73	**0.84/0.86**
A2289_04220	Deltaproteobacteria; Unclassified	599	5-fold?	0.61/0.67	0.62/0.67	0.43/0.51
TDE_2508	Spirochaetota; *Treponema denticola*	455	6-fold; 35aa	0.52/0.6	0.55/0.62	**0.82/0.83**
HMPREF1222_01173	Spirochaetota; *Treponema vincentii*	564	5-fold; 11aa	0.76/0.78	**0.79/0.8**	0.33/0.41
HMPREF1222_01741	Spirochaetota; *Treponema vincentii*	603	5-fold; 11aa	0.64/0.68	**0.71/0.73**	0.29/0.37
Turpa_0960	Spirochaetota; *Turneriella parva*	442	5-fold; 10aa	0.65/0.71	**0.7/0.74**	0.57/0.62
EPJ72_03915	Spirochaetota; *Brachyspira pilosicoli*	495	4-fold?	0.5/0.58	0.24/0.36	0.22/0.32
A2252_05190	Elusimicrobiota; Unclassified	494	5-fold; 10aa	0.69/0.65	**0.8/0.82**	0.63/0.67
A2278_03830	Elusimicrobiota; Unclassified	688	4-fold?	0.7/0.74	0.7/0.73	0.6/0.63
E4H13_05290	Calditrichota; Unclassified	378	6-fold; 22aa	0.61/0.68	0.66/0.7	**0.83/0.85**
Cabys_2908	Calditrichota; *Caldithrix abyssi*	370	6-fold; 21aa	0.4/0.5	0.6/0.65	**0.79/0.81**
Cabys_3254	Calditrichota; *Caldithrix abyssi*	593	5-fold; 10aa	0.58/0.65	**0.7/0.74**	0.64/0.68
D6828_00045	Nitrospirota; Unclassified	449	6-fold; 21aa	0.62/0.68	0.6/0.65	**0.8/0.81**
CBD97_01060	Alphaproteobacteria; *Pelagibacteraceae*	302	6-fold; 25aa	0.52/0.6	0.61/0.65	**0.81/0.83**
EG825_11870	Betaproteobacteria; *Rhodocyclaceae*	428	6-fold; 21aa	0.62/0.68	0.54/0.6	**0.74/0.77**
ENX80_00785	Campylobacterota; *Desulfurella acetivorans*	420	6-fold; 23aa	0.51/0.61	0.63/0.68	**0.77/0.77**
FSU_0230	FCB superphylum; *Fibrobacter succinogenes*	409	5-fold; 12aa	0.73/0.76	**0.84/0.85**	0.75/0.78
FSU_2404	FCB superphylum; *Fibrobacter succinogenes*	434	6-fold; 18aa	0.47/0.58	0.59/0.65	**0.8/0.82**
CMG28_01050	FCB superphylum; Ca. *Neomarinimicrobium*	297	6-fold; 26aa	0.7/0.75	0.55/0.61	**0.82/0.83**
CMG07_05295	FCB superphylum; Ca. *Neomarinimicrobium*	371	6-fold; 23aa	0.64/0.7	0.6/0.65	**0.75/0.78**
enn20_03375	FCB superphylum; Ca. *Neomarinimicrobium*	588	4-fold; 7aa	**0.72/0.75**	0.64/0.69	0.35/0.44
CME24_00930	FCB superphylum; *Gemmatimonadota*	388	6-fold; 23aa	0.64/0.7	0.52/0.58	**0.72/0.75**
CHB002000452	FCB superphylum; *Chlorobiota*	405	6-fold?	0.67/0.72	0.73/0.76	0.75/0.78
GX467_09555	FCB superphylum; Chlorobiota,	604	4-fold; 5aa	**0.8/0.81**	0.67/0.71	0.41/0.48

	Rikenellaceae					
IPM56_03505	FCB superphylum; *Ignavibacteriales*	414	6-fold; 20aa	0.71/0.76	0.68/0.73	**0.86/0.87**
HXY50_09160	FCB superphylum; *Ignavibacteriales*	599	4-fold; 9aa	**0.69/0.74**	0.59/0.65	0.4/0.47
D6800_14570	FCB superphylum; *Zixiibacteriota*	388	6-fold; 20aa	0.71/0.76	0.78/0.81	**0.83/0.85**
TRIP_C90081	FCB superphylum; *Zixiibacteriota*	415	6-fold; 21aa	0.61/0.67	0.6/0.66	**0.82/0.84**
DRQ25_05465	FCB superphylum; *Fermentibacteria*	567	4-fold?	0.76/0.78	0.74/0.76	0.67/0.7
C4574_01495	FCB superphylum; *Latescibacterota*	410	6-fold; 32aa	0.48/0.56	0.7/0.74	**0.82/0.83**
ENV29_01170	Ca. *desantisbacteria*	484	5-fold?	0.53/0.6	0.53/0.6	0.38/0.37
AMJ52_03020	Bacteria division TA06	550	5-fold; 9aa	0.69/0.73	**0.81/0.83**	0.6/0.64
AMJ82_08425	Bacteria division TA06	411	6-fold; 26aa	0.57/0.63	0.57/0.62	**0.6/0.65**
ENX78_07415	Ca. *poribacteria*	374	6-fold; 25aa	0.33/0.45	0.59/0.64	**0.62/0.66**
HUU05_26320	candidate division KSB1 bacterium	599	5-fold?	0.64/0.69	0.66/0.7	0.49/0.54
A2V82_10145	candidate division KSB1 bacterium	588	4-fold?	0.69/0.73	0.68/0.71	0.49/0.54
A2519_13135	Raymondbacteria; Unclassified	450	6-fold; 21aa	0.52/0.61	0.59/0.65	**0.74/0.76**
DRQ10_05220	Hydrothermota; B15_G9	361	6-fold; 21aa	0.59/0.65	0.5/0.57	**0.66/0.67**
CBD58_00495	TMED198 Unclassified	347	6-fold; 20aa	0.43/0.53	0.59/0.65	**0.8/0.82**
DCX15_05615	UBA9089 Unclassified	642	5-fold; 19aa	0.58/0.65	**0.81/0.83**	0.48/0.53
BVY01_02690	Bacterium I07 Unclassified	429	6-fold; 25aa	0.49/0.58	0.71/0.75	**0.83/0.85**
Hore_23180	Bacillota; *Halothermothrix orenii*	561	4-fold; 9aa	**0.71/0.74**	0.56/0.6	0.25/0.33
A7315_03100	Candidatus *Altiarchaeales* archaeon WOR_SM1_79	549	5-fold; 11aa	0.45/0.53	**0.76/0.77**	0.63/0.66

*close scoring alternatives are decided upon by using colabfold dimer to report on angular offset

Supplementary Table 4. BT1926 superfamily members. Features of family members are given, alongside pTM/ipTM Alphafold3[19] scores for oligomer state models.

Gene	Species	Size	State	Tetramer	Pentamer	Hexamer
HMPREF9455_00941	*Dysgonomonas gadei*	244	tetramer	**0.81/0.83**	0.7/0.72	0.54/0.58
H1XQG5, Cabys_3215	*Caldithrix abyssi*	225	?	0.76/0.79	0.76/0.79	0.76/0.78
GX715_16025	Terrabacteria, *Armatimonadota*	195	hexamer	0.35/0.46	0.44/0.5	**0.73/0.76**
CMH57_15440	*Myxococcales*	203	?	0.78/0.8	0.76/0.78	0.73/0.75
EDS67_03010	KSB1 bacterium	229	?	0.73/0.77	0.76/0.78	0.8/0.81
BT1926	*Bacteroides thetaiotaomicron*	288	pentamer	0.7/0.73+	**0.77/0.78**	0.66/0.68
EII40_06770	*Tannerella forsythia*	248	?	0.7/0.74	0.74/0.76	0.77/0.78
AB406_2108	*Riemerella anatipestifer*	248	hexamer	0.5/0.58	0.64/0.68	**0.79/0.8**
DBX24_02045	*Bergeyella cardium*	234	hexamer	0.61/0.67	0.69/0.71	**0.78/0.79**
CHU_0007	*Cytophaga hutchinsonii*	259	hexamer	0.53/0.59	0.64/0.67	**0.73/0.75**
CRI93_11930	*Longimonas halophila*	218	hexamer	0.63/0.68	0.67/0.7	**0.8/0.81**
GWO68_14655	*Pontibacter fetidus*	257	?	0.44/0.52	0.48/0.55	0.48/0.54
GWO68_11565	*Pontibacter fetidus*	213	hexamer	0.73/0.76	0.78/0.79	**0.83/0.84**
LX69_01076	*Breznakibacter xylanolyticus*	278	pentamer ?	0.66/0.69	0.76/0.77	0.74/0.75
HUU32_21835	*Calditrichaceae*	225	?	0.72/0.75	0.73/0.75	0.44/0.51
Phep_3878	*Pedobacter heparinus*	237	hexamer	0.75/0.78	0.73/0.75	**0.83/0.84**
HMPREF9448_01165	*Barnesiella intestinihominis*	229	hexamer	0.61/0.65	0.55/0.6	**0.81/0.82**
R7P9Y3	*Prevotella* sp. CAG:617	218	?	0.73/0.75	0.71/0.73	0.72/0.74
E6K75_10110	*Eisenbacteria*	253	tetramer	**0.75/0.78**	0.7/0.73	0.7/0.72
SapgrDRAFT_1972	*Saprospira grandis*	284	nonamer	tetramer<br>0.56/0.61<br>heptamer<br>0.61/0.64<br>decamer<br>0.66/0.67	pentamer<br>0.58/0.63<br>octamer<br>0.65/0.67	hexamer<br>0.52/0.57<br>nonamer<br>**0.82/0.82**
L21SP5_03224	*Salinivirga cyanobacteriivorans*	293	nonamer?	tetramer<br>0.71/0.74<br>heptamer<br>0.68/0.7<br>decamer<br>0.77/0.78	pentamer<br>0.69/0.72<br>octamer<br>0.74/0.76	hexamer<br>0.66/0.69<br>nonamer<br>0.75/0.77
COB30_01920	*Pseudomonadota, Ectothiorhodospiraceae*	198	pentamer	0.73/0.77	**0.82/0.83**	0.77/0.78
HOP24_10745	*Pseudomonadota, Sideroxydans*	181		**0.88/0.89**		
HNR50_000903	*Spirochaeta isovalerica*	204	hexamer	0.55/0.62	0.63/0.67	**0.8/0.82**
BZG01_18965	*Labilibaculum manganireducens*	199	tetramer	**0.77/0.8**	0.69/0.73	0.61/0.66
CGC59_13065	*Capnocytophaga sputigena*	228	Nonamer?	tetramer<br>0.15/0.3<br>heptamer<br>0.6/0.63	pentamer<br>0.59/0.63<br>octamer<br>0.63/0.66	hexamer<br>0.56/0.62<br>nonamer<br>0.67/0.69

*close scoring alternatives are decided upon by using colabfold dimer to report on angular offset
+models as pentamer (rather than closed tetramer), missing one subunit

Supplementary Table 5. Bacterial strains used in this study.

Strains	Description	Resistance	Source
**GL655**	MG1655. WT *E. coli* strain	/	Laloux lab collection
**GL1843**	MG1655 + pTHV037-*lpoB-mCherry_msfgfp-glpT.* This strain was called $MG1655_{ER}$ for MG1655 envelope reporter	Amp	This study
**GL1890**	$MG1655_{ER}$ + pBAD18-*popA*	Amp/Chlor	This study
**GL2132**	$MG1655_{ER}$ + pBAD18-$ompF_{E. coli}$	Amp/Chlor	This study
**GL2191**	MG1655 + pBAD18-$bd0427_{Bb}$-$_{Inter}$*mCherry*	Chlor	This study
**E. coli DH5α**	E. coli cloning strain (*fhuA2Δ(argF-lacZ)U169 phoA glnV44 Φ80Δ(lacZ)M15 gyrA96 recA1 relA1*)	N/A	New England Biolabs (C2987)
**E. coli TOP10**	E.coli cloning strain (F- mcrA Δ(mrr-hsdRMS-mcrBC) Φ80lacZΔM15 Δ lacX74 recA1 araD139 Δ(araleu)7697 galU galK rpsL (StrR) endA1 nupG)	Str	Thermo Fisher (C404003)
**E. coli S17-1**	E. coli strain (thi, pro, hsdR-, hsdM+, recA; integrated plasmid RP4- Tc::Mu-Kn::tn)	Kan	Hanahan D., 1983[3]
**E. coli S17-1: pZMR100**	E. coli strain containing the plasmid pZMR100 (kanR)	Kan	Rogers M, et al., 1986[4]
B. bacteriovorus HD100	*B. bacteriovorus* Type strain, genome-sequenced, wild-type	N/A	Rendulic S, *et al.*, 2004[5]
B. bacteriovorus HD100 Bd0427_A9C_S267C_SXO	HD100 containing a single-crossover, full length Bd0427 with the point mutations A9C and S267C	N/A	This study
B. bacteriovorus HD100 *Δbd0427*	*B. bacteriovorus* containing an in-frame unmarked deletion of *bd0427*	N/A	This study

Supplementary Table 6. Primer and plasmid information.

Primer	Sequence (5' to 3')
oGL1487	GCTAGCCCAAAAAAACGGGTATGG
oGL1488	GGTACCCGGGGATCCTCTAG
oGL1727	CGTTTTTTTGGGCTAGCAGGAGGTATTACACCATGGATTTAAAAGTTCATAAGATTGCAACG
oGL1728	GGATCCCCGGGTACCTTAGAAAGTGTAAGTGTAAGCAACTTGAGAG
oGL1765	CGTTTTTTTGGGCTAGCATGATGAAGCGCAATATTCTGGC
oGL1766	GGATCCCCGGGTACCTTAGAACTGGTAAACGATACCCACAG
oGL2256	GAAAGTGTAAGTGTAAGCAACTTGAGAG
oGL2261	GACTGTTCGTGATGCCGGATCCGTGAGCAAGGGC
oGL2262	CAGCGAAAGCTGGATTGGATCCCTTGTACAGCTCGTCCATGCC
Bd0427-ko-up-F-2	TAAAACGACGGCCAGTGCCAATAAGTGGACAAAGTGGC
Bd0427-ko-up-R-2	CTTAGAAAGTTTTCATTGTTGCATCCTTTCGTTT
Bd0427-ko-dw-F-2	AACAATGAAAACTTTCTAAGACCTAGTCTTAAAGTTTAAGAA
Bd0427-ko-dw-R-2	ACAGCTATGACATGATTACGTAATTTAAGTTCCCCCAAAC
Bd427mCh_DXO_UP_F	cgttgtaaaacgacggccagtgccaGGAACTAAAGCTACTTCTGCTG
Bd427mCh_Link_R2	TGCTCACCATACCACCGCTGCCACCGCCGCCGCTACCGCCGAAAGTGTAA
427mCherry_Link_F	TTACACTTTCGGCGGTAGCGGCGGCGGTGGCAGCGGTGGTATGGTGAGCA
427mCherry_R	ttaagactaggtcTTACTTGTACAGCTCGTCCATG
Bd427_FLANK_DN_F	gctgtacaagtaaGACCTAGTCTTAAAGTTTAAGAACTC
Bd427_FLANK_DN_R	ggaaacagctatgaccatgattacgAATGTGAGCTTCATACCAAC
Bd427mut_UP_F	CGTTGTAAAACGACGGCCAGTGCCACGCATAGTTCAGGCGGGTC
Bd427mut_UP_R	CACGTGCTTTAGAAGCCATTGCTGCTGGAGC
Bd427mut_MID_F	AGCAGCAATGGCTTCTAAAGCACGTGTAGAGG
Bd427mut_sxo_R	GGAAACAGCTATGACCATGATTACGTTAGAAAGTGTAAGTGTAAGCAAC
Bd0427_p28_F	cccctctagaaataattttgtttaactttaagaaggagatataccatgtctaaagcacgtgtagaggctttg
Bd0427_p28_R	agccggatctcagtggtggtggtggtggtgctcgagttagaaagtgtaagtgtaagcaacttg

Plasmid	Description	Source
pK18*mobsacB*	Suicide vector (kanR, *lacZa*, *sacB*) used for crossovers into the *B. bacteriovorus* genome	Schafer *et al.* 1994[1]
pAKF56-mCherry	Template for *mCherry* gene.	Fenton *et al.* 2010[2]
pET28-Bd0427_A9C_S267C	Truncated Bd0427 starting at residue A20 with point mutations A9C and S267C	This study
p0427_A9C_S267C_SXO	Full length Bd0427 with point mutations A9C and S267C (single-crossover)	This study
pdelta0427	Upstream and downstream fragments of *bd0427* gene for unmarked gene deletion	This study
p0427-mCh_DXO	Full length Bd0427-mCherry fusion (double-crossover)	This study

Supplementary References

1. Schafer A, Tauch A, Jager W, Kalinowski J, Thierbach G, Puhler A. Small mobilizable multi-purpose cloning vectors derived from the Escherichia coli plasmids pK18 and pK19: selection of defined deletions in the chromosome of Corynebacterium glutamicum. *Gene* **145**, 69-73 (1994).
2. Fenton AK, Kanna M, Woods RD, Aizawa SI, Sockett RE. Shadowing the actions of a predator: backlit fluorescent microscopy reveals synchronous nonbinary septation of predatory Bdellovibrio inside prey and exit through discrete bdelloplast pores. *J. Bacteriol.* **192**, 6329-6335 (2010).
3. Hanahan D. Studies on transformation of Escherichia coli with plasmids. *J. Mol. Biol.* **166**, 557-580 (1983).
4. Rogers M, Ekaterinaki N, Nimmo E, Sherratt D. Analysis of Tn7 transposition. *Mol Gen Genet* **205**, 550-556 (1986).
5. Rendulic S, *et al.* A predator unmasked: life cycle of *Bdellovibrio bacteriovorus* from a genomic perspective. *Science* **303**, 689-692 (2004).
