# Supplementary figures and images for: A porin-like protein used by bacterial predators defines a wider lipid-trapping superfamily
Source: Nat Commun. 2025 Jul 5;16:6213. doi: 10.1038/s41467-025-61633-0 (PMC12228718; doi:10.1038/s41467-025-61633-0)

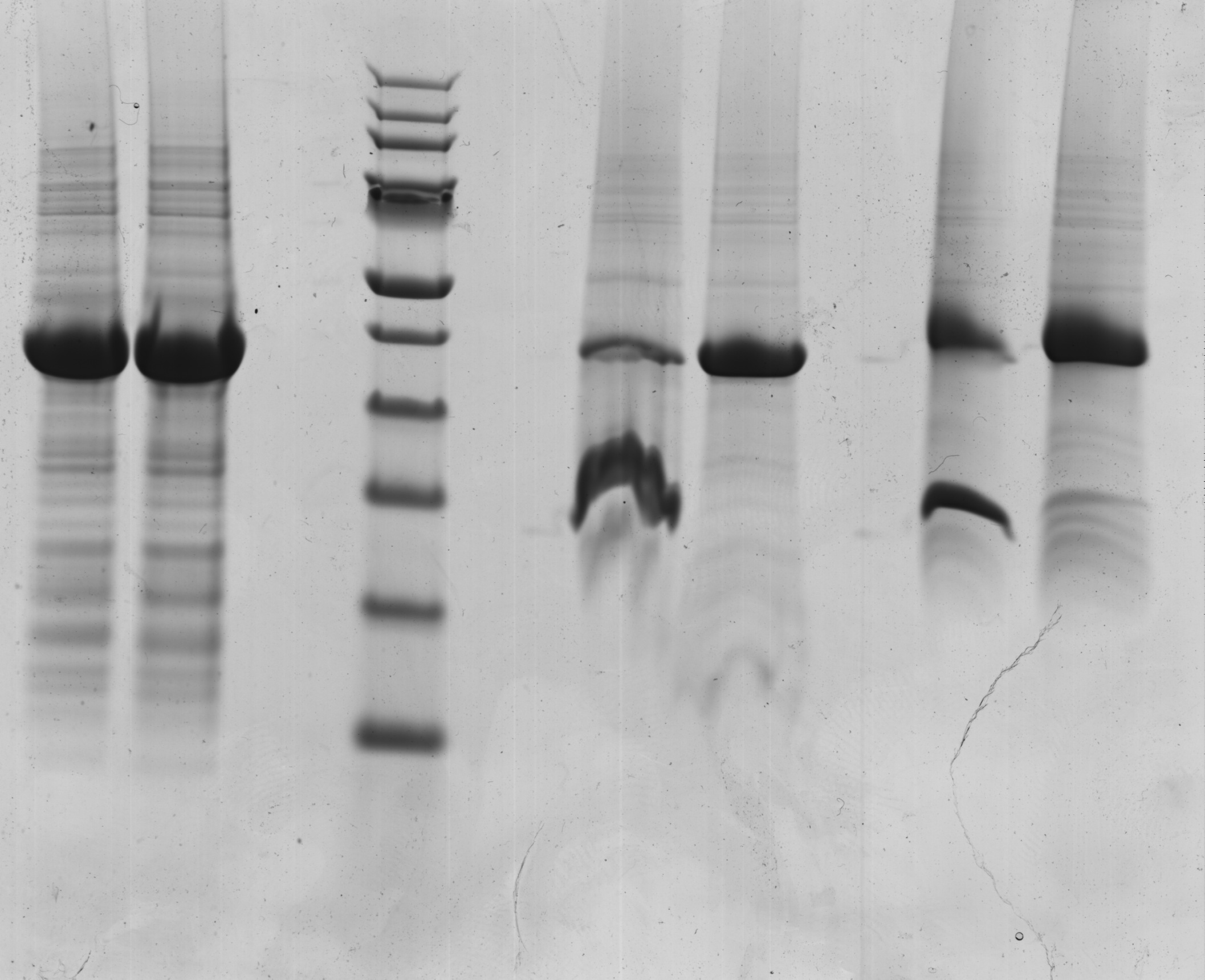

Supplement: Supplementary file 4 — Source Data [file 41467_2025_61633_MOESM4_ESM.zip › source_data/supplementary_figure1_gel_uncropped.tif]

Figure. 5b

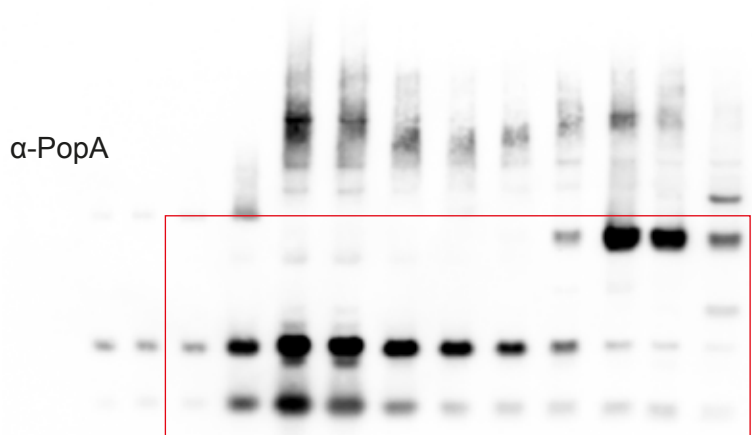

$\alpha$ -Lpp

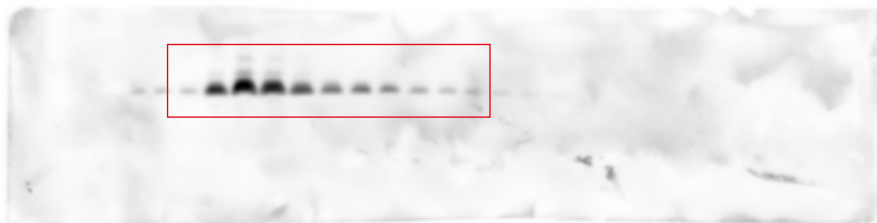

$\alpha$ -LolC

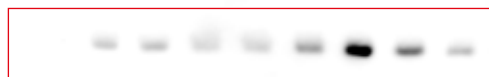

Supplement: Supplementary file 4 — Source Data [file 41467_2025_61633_MOESM4_ESM.zip › source_data/Unpcropped scans_Figure5.pdf]
